# Computer Simulation of Assembly and Co-operativity of Hexameric AAA ATPases

**DOI:** 10.1371/journal.pone.0067815

**Published:** 2013-07-15

**Authors:** Doan Tuong-Van Le, Thomas Eckert, Günther Woehlke

**Affiliations:** Department of Physics E22 (Biophysics), Technische Universität München, Garching bei München, Germany; Wake Forest University, United States of America

## Abstract

AAA ATPases form a functionally diverse superfamily of proteins. Most members form homo-hexameric ring complexes, are catalytically active only in the fully assembled state, and show co-operativity among the six subunits. The mutual dependence among the subunits is clearly evidenced by the fact that incorporation of mutated, inactive subunits can decrease the activity of the remaining wild type subunits. For the first time, we develop here models to describe this form of allostery, evaluate them in a simulation study, and test them on experimental data. We show that it is important to consider the assembly reactions in the kinetic model, and to define a formal inhibition scheme. We simulate three inhibition scenarios explicitly, and demonstrate that they result in differing outcomes. Finally, we deduce fitting formulas, and test them on real and simulated data. A non-competitive inhibition formula fitted experimental and simulated data best. To our knowledge, our study is the first one that derives and tests formal allosteric schemes to explain the inhibitory effects of mutant subunits on oligomeric enzymes.

## Introduction

### Expression from Two Alleles

Enzymes often work in oligomeric assemblies with multiple, interacting subunits. The blueprints for the primary structure of enzymes lie in the genomes of organisms. Most eukaryotes are diploid, meaning that two copies of the genome are present in each cell, one from each parent. Hence, two different alleles of each gene can be present. For most (but not all) human genes the expression levels of the two alleles are similar, and no allele-specific expression is found [1]. For this majority of cases, oligomeric enzymes are composed of proteins expressed from different alleles. In normal cases, this does not have any consequences for the organism because both alleles are very similar and usually both functional. However, in pathological cases defective genes can have a dominant-negative effect on the intact allele of the gene. Although there are several mechanisms that can lead to dominant-negative inheritance among them haplo-insufficiency, aggregation of the mutated gene product, complex genetic feedback circles we focus here on cases where the gene products of the intact and the defective allele co-assemble into a protein complex, and the mutant gene product inhibits the proper function of the wild type gene product.

### Hereditary Spastic Paraplegia and Dominance of Spastin Mutations

Our current study is inspired by the study of the microtubule-severing enzyme spastin, which is encoded by the human SPAST or SPG4 gene. SPG4 has been identified in families suffering from hereditary spastic paraplegia (HSP), a disease that typically manifests itself between the second to fourth life decade by a progressive weakness of the lower limbs. The ‘Online Mendelian Inheritance in Man’ database (http://omim.org/entry/604277?search=spastin highlight = spastin) lists 22 disease-related allelic variants of the SPG4 gene, among them at least ten that lead to single amino acid changes. It is likely (and even has been shown in some cases) that the disease-related variant is expressed along with the wild type allele, suggesting that the dominant-negative effect of these mutations emerges at the protein level. The same is true for SPG10, another HSP gene that encodes the kinesin-1 type microtubule motor protein KIF5A. Several SPG10 mutations are known from HSP patient families that destroy the motor function of KIF5A, suggesting that their dominant-negative effect is imposed by an altered gene product. In vitro and in vivo studies have supported this notion [2], [3].

### Combinatorics of Enzyme Oligomerization

**Figure 1:**
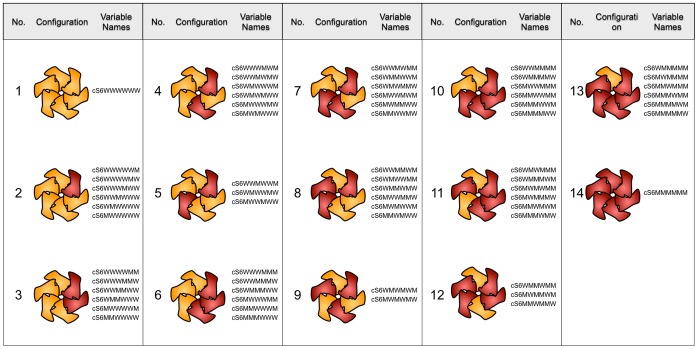
Possible configurations of subunits originating from different alleles. The subunits encoded by allele 1 (e.g. wild type, yellow) and allele 2 (e.g. inactive mutant, red) are shown. The number is an arbitrary identifier of each conformation. The ‘Variable Names’ 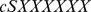 (X either W or M, for wild type or mutant) signify the concentrations of the particular hexamer conformation in a simulation run. In the simulation, rotational permutations of a given conformation (represented by the sequence of the letters M and W) arise, and are summed up to give the concentration of the particular hexamer conformation.

To understand the way in which spastin and KIF5A mutations invoke HSP in more detail, it is important to know that both enzymes are functional as oligomers. KIF5A is active as a homo-dimer, spastin as a homo-hexamer. As argued above, in heterozygous patients both are likely to be composed of intact and ‘defective’ subunits. Kinesin heavy chains form stable dimers that do not exchange at an observable rate. Assuming equal expression and protein production from both alleles, one can easily calculate the probabilities of finding all combinations of wild type and mutant subunits from a binomial probability distribution (namely 1∶2:1 (wt+wt : wt+mutant : mutant+mutant) if equal amounts of wild type and mutant are present). The situation is relatively easy because allosteric influences of one motor head on its partner have been investigated extensively, and for several mutations the kinetic intermediate that is affected is known [2], [4]. Spastin displays a far more complex behavior. It assembles and disassembles dynamically, and forms hexameric rings. To appreciate the tremendous increase of complexity in comparison to kinesin, one has to consider the following facts: (i) Six subunits per functional unit allow many more mixed states, with one, two, …, six mutant subunits per ring. The number of mutated subunits per spastin hexamer ring can still be calculated assuming a binomial distribution. In addition, however, there are several configurations of rings with a given number of mutant subunits (Fig. 1). For examples, there are three ways of arranging two mutant subunits in a hexameric ring (configurations 3–5 in Fig. 1). Due to the ring shape of enzyme structures, identical configurations can occur as rotational permutations. They cannot be mirrored because there is an ‘upper’ and a ‘lower’ side of the ring [5]. The symmetry properties of these configurations are important because the comparison with other AAA ATPases suggests that the activity of one subunit requires the neighboring subunit to provide an arginine residue for the catalytic process [6]–[9]. In agreement, patient families with a spastin R499C mutation suffer from HSP [10], [11]. The arginine finger comes from (only) one oriented neighbor. Therefore, it is important to know which neighbor is intact or defective. The increase in complexity is also due to (ii) the dynamic assembly behavior, which makes it necessary to consider assembly and disassembly rates that are different for wild type and mutant subunits. In the case of kinesin, the dimeric structure is stable over periods much longer than the catalytic cycle, and hence negligible. The assembly rate is also unaltered by mutations, because all known mutations are in the catalytic or the cargo-interaction domain, and not in the neck domain, which is responsible for the coupling of the motor domains [2], [12], [13]. Another well-studied example is GroEL, which is also stable over the course of multiple catalytic turnovers [14]. In contrast, spastin is mostly monomeric, and assembles into hexamers only for short periods of time [5], [15], [16]. In fact, only one mutant in the ATP-binding cleft the E442Q (human spastin numbering) point mutant in the Walker B motif has been observed to form oligomers that are stable enough to be observed in gel filtration, analytical ultracentrifugation, cross-linking and SAXS experiments [5], [15], [16]. Another observation emphasizes the importance of hexamer assembly rates: an artificial, dimeric spastin construct behaved as if it were fully activated by microtubules (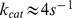 instead of 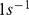). Importantly, microtubules were shown to facilitate formation of higher oligomeric states, suggesting that oligomerization and catalytic turnover are intricately convolved [17]. As a third complication, (iii) spastin is an allosteric enzyme with a co-operative mechanism. Kinetic studies with ATP analogs and inactive spastin mutants showed that at least two of the six subunits influence each other strongly [16]. This was concluded from quantitative kinetic experiments using ATP and the substrate analog ATP-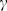
S, and by the dominant-negative effect of inactive mutant subunits. Still, the allosteric coupling in spastin hexamers is much less clear than that of, for example, kinesin and GroEL, which are also allosteric enzymes. Their co-operativity has been characterized in series of publications [14], [18]. These three arguments show how complex oligomerization and functionality are intertwined, and how difficult it is to predict dominant-negative effects of mutant subunits on wild type protein in mixed oligomers. Although there are many ‘classical’ theories on allosteric mechanisms that deduce allosteric properties from (homotropic or heterotropic) ligand effects [19]–[22], there are only few studies that address the question how mutant subunits can exert a dominant-negative effect from a conceptional and systematical point of view. Several publications on different AAA ATPases reported the inhibitory effect of mutants in mixed wild type-mutant oligomers, and identified the observations as allosteric effects [23]–[28]. A common scheme underlying these specific cases, however, has not been discovered yet [29]. The mathematical description of hexamer assembly leads to non-linear systems of equations and cannot be solved analytically (Model and Methods). Such systems can be investigated by numerical integration or simulations. We approached the problem by Monte-Carlo simulations that model the behavior of wild type spastin, and mixtures of wild type and mutant spastin, because numerical integrations on ensembles of finite sizes that are allowed form oligomers of limited sizes have been shown to possibly lead to inaccurate results [30].

## Results

To interpret the inhibition pattern of mixtures of wild type and mutated spastin we first show that the assembly pathway kinetics of hexameric rings is an important determinant for the steady state ATPase turnover, before we investigate alternative allosteric schemes that can explain experimental observations. We finally demonstrate that one of these schemes describes the observed behavior best. Note that we will use the term ‘pathway’ in the context of oligomer assembly, the term ‘scheme’ if we refer to inhibition networks and patterns.

### Oligomerization and Activity of Wild Type Spastin

**Figure 2:**
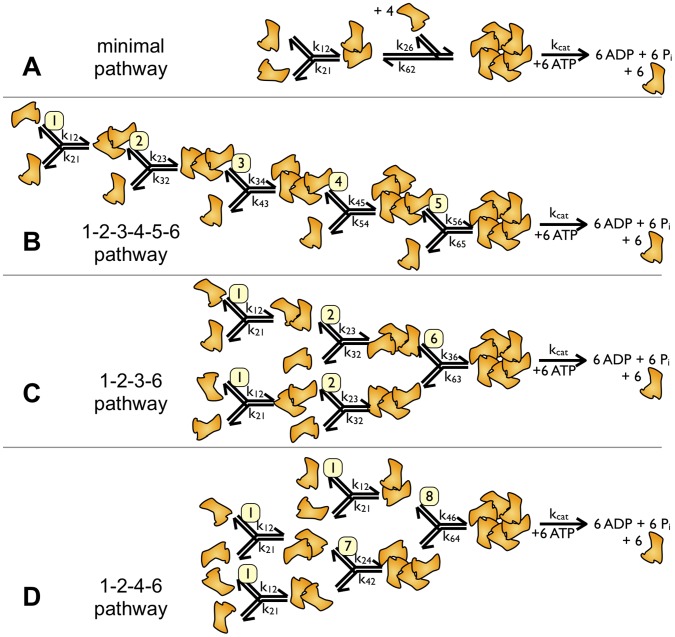
Assembly pathways. The figure shows four ways of assembling hexameric ring structures from monomers. Panel A is a scheme that treats all steps after dimer formation as one collective step. The reactions leading to a hexamer are treated as reversible reactions (rates along the arrows), the catalytic step as irreversible (rate 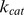). Panel B fills in the missing steps from dimer to hexamer by sequential addition of single subunits. We term the pathway 1–2–3–4–5–6 to indicate the oligomeric intermediates. Panels C and D show pathways in which partially assembled intermediates are allowed to react with each other. In panel C, trimeric intermediates are allowed to react with each other (hence 1–2–3–6 pathway), in panel D dimers are allowed to react with each other and with tetrameric intermediates (1–2–4–6 pathway). Numbers with yellow background enumerate reactions of different intermediates (the 9th reaction of dimers with trimers does not occur in the displayed pathways).

In experiments, dimeric spastin assemblies, but no higher oligomeric states, have been detected [5], [16]. The evidence that hexamers still are the active form of the enzyme are indirect. (i) Inhibition studies show that the addition of inactive mutants to wild type spastin slows down the ATP turnover per wild type subunit [16]. (ii) Moreover, structural investigations of mutant protein and analogies to other AAA ATPases indicate that spastin forms hexameric ring structures that probably represent the active form of the enzyme [5], [15], [16], [31], [32]. Hence, at saturating ATP concentrations, the assembly pathway shown in figure 2 is the most basic one (Fig. 2A). 

The catalytic step ATP turnover occurs at the rate 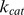 after the hexamer has been formed, unless the hexamer disassembles with the reverse rate k62. Since our model assumes saturating ATP concentrations, the ATP binding step does not need to be implemented as a bi-molecular reaction, and might occur before or after hexamer formation. It is an important assumption of our model, however, that the disassembly step k62 is not accompanied by product (ADP and phosphate) release. Figure 2A treats the steps between dimerization and hexamerization as one collective step. As the simultaneous encounter of five molecules is negligible, intermediate oligomers must play a crucial role in the progress of hexamerization. In reality, subunits could join the partially assembled oligomer one by one in a sequential manner (Fig. 2B). For a hexamer, this would involve five different reactions (1+1, 2+1, 3+1, etc.; termed 1–2–3–4–5–6 assembly pathway). However, in homo-hexamers, reactions of partially assembled intermediates among each other are to be expected [33]. This reduces the number of different reactions that lead to full assembly. Note that the number of steps is always five but the number of different reactions may be less than five (Fig. 2). The pathways involving the least number of different reactions are shown in figure 2. They involve either dimeric and trimeric intermediates (termed 1–2–3–6 pathway, Fig. 2C), or dimeric and tetrameric intermediates (1–2–4–6 pathway, Fig. 2D). In principle, mixed pathways with up to nine reactions are possible (the ninth reaction, trimer plus dimer, is missing in figure 2). However, the 1–2–3–6 and the 1–2–4–6 pathways have a special feature: They require the least accumulation of different intermediate oligomer pools. This is important because all forward rates are concentration-dependent, and in practice only occur if a sufficient amount of the reaction partners is available.

**Figure 3:**
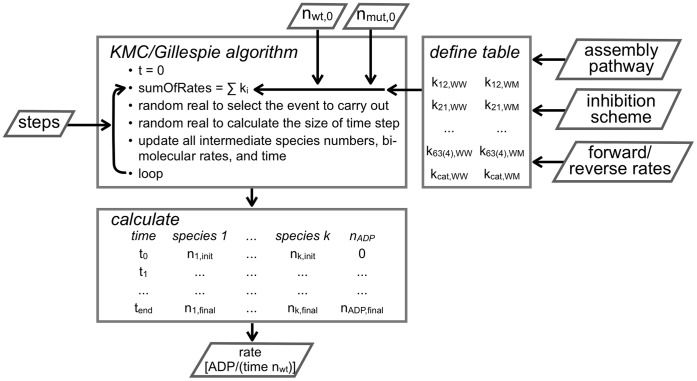
Flow chart of simulation. The figure shows the computational implementation of the Monte Carlo simulation, its input parameters, and the output produced.

**Figure 4:**
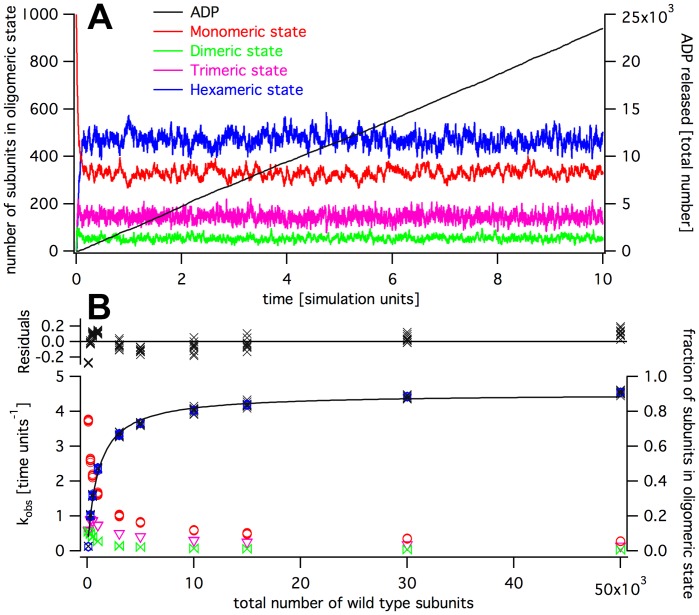
Simulation of wild type activity according to the 1–2–3–6 assembly pathway. Panel A: Time trace of cADP, and intermediate oligomer concentrations. Panel B: Concentration dependence of activity with a Michaelis-Menten curve fit.

**Figure 5:**
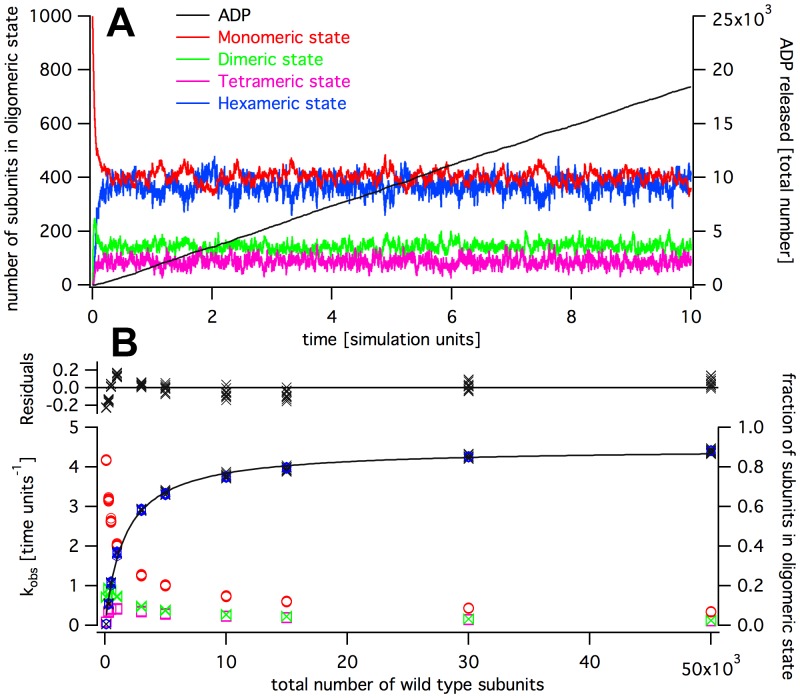
Simulation of wild type activity according to the 1–2–4–6 assembly pathway. Panel A: Time trace of cADP, and intermediate oligomer concentrations. Panel B: Concentration dependence of activity with a Michaelis-Menten curve fit.

A quantitative description of the oligomerization process, however, is complicated. From figure 2, one can derive differential equations that describe the temporal development of the system (Model and Methods). As the concentration of free enzyme goes into the rate equations with the first and second power (depending on the step), a system of five coupled non-linear equations arises that cannot be solved analytically. To describe the biochemical reactions properly, and to be able to set up new experiments, we used a Kinetic Monte-Carlo simulation for our system [34]. We implemented the simulation as described in the Model and Methods section (Fig. 3). In a first step, we modeled the dependence of the ATP turnover rate on the spastin concentration (Fig. 4 and 5). In experiments, the activity has been shown to saturate at high enzyme concentrations [16]. As an additional requirement to match the experimental observations, we chose the assembly parameters to keep the equilibrium monomer concentration as high as possible, and to keep the number of dimers above the number of trimers (tetramers). 

These requirements were fulfilled when the dimerization step was slow, the following oligomerization steps were faster, and the catalytic step were slightly higher than the observed maximal turnover rate, 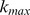 (Tab. 1). Note that we define here 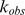 as the observed substrate turnover rate under a specific condition, 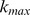 as the extrapolated maximal rate, and 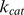 as the catalytic constant (as indicated in figure 2) used in the simulation. For the following simulations, we used the default set of parameters given in table 1. The simulation gave reproducible traces for a given set of parameters (Fig. 4B and 5B show the data of ten simulation runs for each point). 

**Table 1:** Default parameter values.

reaction	forward rates	reverse rates
2 monomers 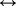 dimer	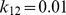	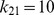
dimer+monomer 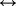 trimer	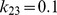	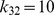
2 dimers 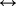 tetramer	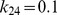	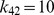
2 trimers 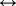 hexamer	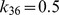	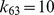
tetramer+dimer 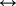 hexamer	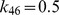	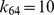
catalytic step (hexamer 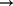 6 monomers)	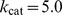	n.a. (irreversible)

Standard Rates Used in the Wild Type Simulation.

**Figure 6:**
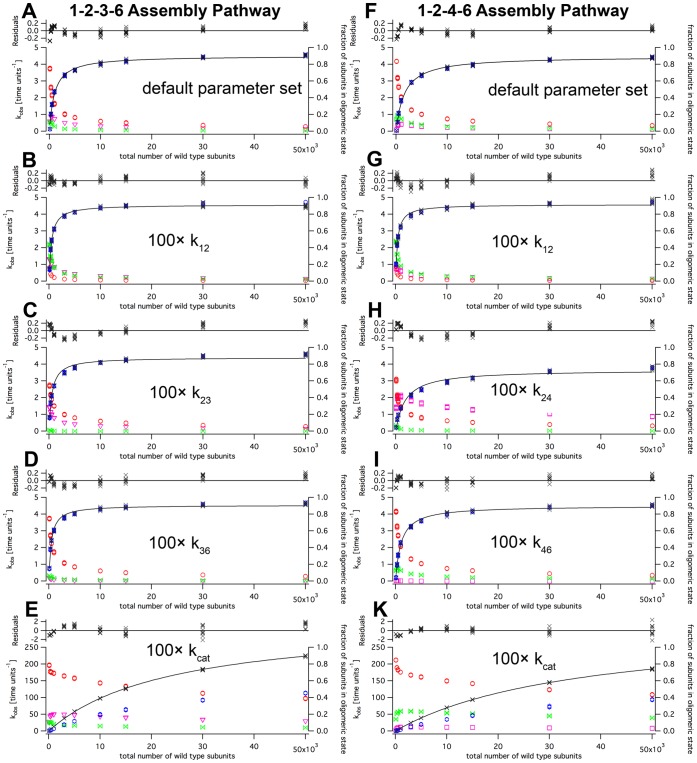
Dependence of wild type steady state kinetics on the choice of parameters. Panels A–E show simulations according to the 1–2–3–6 pathway, F–K the 1–2–4–6 pathway. Data on panels A and F was created with the default parameter set. In panels B and G, the dimerization step, 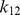, was accelerated 100-fold. In panel C the rate of timer formation from monomers and dimers, 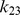, was increased 100-fold, in H the rate of tetramer formation, 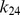. Panels D and E show the effect of 100-fold increased hexamer formation rates, 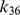 (1–2–3–6 pathway) and 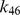, (1–2–4–6 pathway). Finally, panels F and K display the effect of accelerated 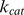 values. The left axes show kobs, drawn is black crosses in the graph, the right axes the frequency of subunits occurring in specified intermediates (red balls monomers, green double-triangles dimers, magenta triangles timers, magenta squares tetramers, blue hexagons hexamers). The data are fitted with a Michaelis-Menten curve, above the graphs the residuals are given.

The observed and simulated turnover rates (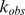) depended on the total enzyme particle number. We performed our simulation with varying total particle numbers, ten times for each condition (Fig. 6). Again, the replicates produced very similar time traces. A plot of the observed turnover rate, 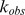, against enzyme particle number followed approximately a Michaelis-Menten kinetics. We therefore fitted the steady state data with equation 8 (Model and Methods). The residuals displayed a systematic deviation, showing that the Michaelis-Menten model does not describe the system accurately. This is not unexpected because the oligomerization is not pseudo-first order, and involves reactions of higher order than assumed in the Michaelis-Menten model. Still, the half-maximal activation constant calculated from the Michaelis-Menten fit, 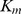, lay close to the particle number at which 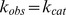 (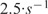 under default parameter settings) was reached. In the 1–2–3–6 assembly pathway with standard parameters, we found 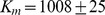 particles, in the 1–2–4–6 assembly pathway 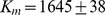 particles. A pseudo-first order approximation (equation 11) predicted 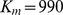 particles, slightly lower than found in simulations (Tab. 2). 

**Table 2:** Fitted parameter values of pure wild type simulated models, and pseudo-first order prediction.

parameter set		1–2–3–6		pseudo- mono–molecular		1–2–4–6	
	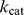	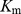	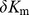	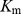	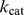	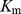	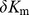
default	4.5	1008	25	990	4.5	1645	38
100 *k*_12_	4.6	451	7	9.9	4.6	346	9
100 *k*_23(24)_	4.4	557	17	504	3.7	1858	77
100 *k*_36(46)_	4.5	458	9	990	4.5	1060	22
100 *k*_cat_	330	23846	160	991	318	35901	271

Response of the model on parameter variation.

Along with the 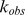 we plotted the fraction of wild type molecules present in a specific oligomeric state in dependence of the total particle number (Fig. 4B and 5B, right axis). The distribution showed that the above requirements were fulfilled over a large concentration range. As the model assumed hexamers to be the only catalytically active form, the catalytic turnover, 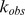, was directly proportional to the number of wild type subunits in hexamers. 

These plots allowed a comparison of the 1–2–3–6 and the 1–2–4–6 assembly pathways (Fig. 6). The 1–2–3–6 assembly pathway produced a steeper dependence of 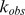 on the particle number, with a lower half-maximal activation constant. Moreover, the 1–2–3–6 assembly pathwayfavored trimers at the expense of the dimeric population. Concomitant with the relatively large trimer population, the number of free monomers in the 1–2–3–6 pathway was lower, showing that trimer formation is a key step in this assembly pathway. In contrast, the 1–2–4–6 pathway produces a fraction of dimeric intermediates larger than that of tetramers. This is because the 1–2–4–6 assembly pathwaycontains two reactions that are driven by a high concentration of dimers. 

To get deeper insight into the dependencies of equilibrium concentrations (and thus 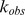) on the oligomerization rates, we increased the rates 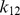, 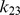, 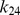, 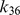, 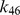 and 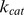 by a factor of 100 (Fig. 6). Not unexpectedly, increasing the dimerzation rate 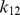 in the 1–2–3–6 assembly pathway increased the number of dimers (Fig. 6C). In turn, trimer formation was favored, and the pool of monomers was reduced. The half-maximal activation constant was reduced from 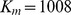 to 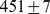 particles, more than two-fold. The pseudo-first order formula predicted 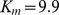 particles. This large discrepancy demonstrates the crucial importance of the initial second order step. 

The 100-fold increase of 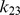 also affected the free monomer number due to a flow of monomers into trimers (Fig. 6E). At the same time, it diminished the pool of subunits in dimers almost completely. The half-maximal activation constant was reduced from 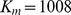 to 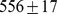 particles, a little less than two-fold compared to the default parameter set. The calculated pseudo-first order value was 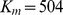. 

Obviously, the increase of rate 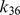 will lead to a reduced amount of trimers (Fig. 6G). Moreover, it will create a situation where dimerization is rate-limiting at low total particle numbers. As suspected from these considerations, the simulations produced curves with a half-maximal activation constant which is a little less than half of the default situation, and the 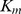 changed from 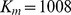 to 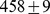 particles. The calculated pseudo-first order yields 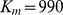 particles. 

As in the 1–2–3–6 assembly pathway, increasing the dimerization rate, 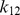, in the 1–2–4–6 pathwayreduced the number of free dimers in equilibrium at the expense of free monomers (Fig. 6D). The half-maximal activation constant was 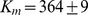 particles instead of 1645 particles in the default parameter set. The calculated pseudo-first order value was 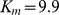 particles. With a 100-fold increased 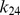 the half-maximal activation constant was largely unaffected (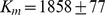 instead of 1645 particles; pseudo-first order 504 particles; Fig. 6F). A 100-fold higher 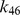 did not lead to major changes, either (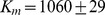 instead of 1645; pseudo-first order 990 particles; Fig. 6H). At low particle numbers, the pool of tetramers was strongly reduced. Over the entire concentration range, the number of free tetramers became rate-limiting. Together, these variations demonstrate that large deviations to the pseudo-first order approximation occur when rates of steps with quadratic dependencies are altered. 

Increasing the 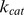 in either assembly pathway to 500, led to a of less than 2/3 of 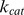, and a huge 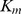 (Fig. 6K). In this case, the pool of hexamers decomposed mainly through the hydrolysis step, making the forward oligomerzation steps rate-limiting.

### Allosteric Basis of Inhibition by Mutant Subunits

In biochemical assays it has been shown that inactive mutant subunits of AAA proteins inhibit the activity of wild type enzymes, demonstrating that wild type and mutant proteins can form mixed oligomers in vitro [16], [24], [26], [27], [35]. In addition, artificial concatemer constructs revealed the importance of different mixed wild type-mutant hexamer configurations [25]. Still, the experimental results are difficult to interpret because the assembly pathways involve a large number of intermediates and combinations, and are based on assumptions that have not been formulated explicitly, nor tested rigorously for global consequences in computer simulations. 

We therefore extended our Kinetic Monte Carlo simulation, and allowed the incorporation of mutant subunits along with wild type (Fig. 1). In our simulations, the mutant was always considered to be unable to hydrolyze ATP (inactive), as shown experimentally for a number of AAA protein point mutants. The mutant’s effect was assumed to originate from the allosteric coupling between the subunits of the hexameric ring, and the activity of wild type subunits in a mixed oligomer modulated by the presence of mutant(s). Accordingly, wild type subunits were allowed to have a ‘normal’ turnover rate, 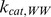, and a reduced, ‘basal’ rate, 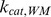, which was manifested in the presence of mutant subunits in the hexameric ring. 

**Figure 7:**
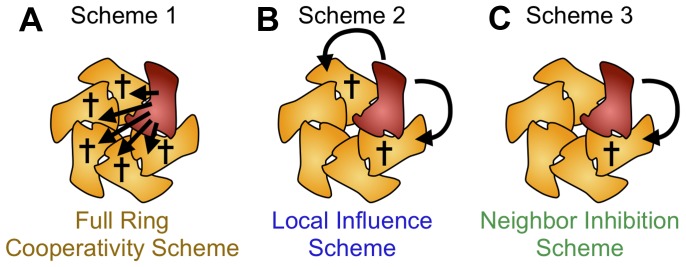
Inhibition schemes. The figure shows three reasonable ways how inactive mutant subunits could inhibit the hexameric assembly. (A) If all subunits were strictly coupled and the activity of each subunit depended on an entirely intact surrounding, any inactive mutant subunit would inhibit all other subunits (scheme 1, full ring cooperativity). (B) If there were only a local effect on the neighboring subunits, only the two neighbors would be inhibited (scheme 2, local influences). (C) The inhibition might be even more specific and directed to only one of the two neighbors (scheme 3, neighbor inhibition). Table 3 was derived from these schemes considering all possible configurations (Fig. 1).Not shown: for the simulation, the affected subunit does not necessarily need to be the neighbor of the mutated subunit but could also lie in the opposing position. Additional schemes not considered here are discussed in the text.

There are different, alternative inhibition schemes to model the allosteric modulation of 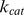 (Fig. 7). In several publications it has been assumed that one single defective subunit per hexamer can abolish, or reduce the catalytic activity of the remaining active subunits to the lower ‘basal’ rate (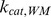) [26]. This assumption resembles Monod-Wyman-Changeux’s model of themutually exclusive existence of ‘tensed’ (T) and ‘relaxed’ (R) states in an oligomer, which, however, does not make predictions on effects of mutant subunits [36]. As it is not the only possible explanation, we are considering here the following alternative inhibition schemes (Fig. 7 and Tab. 3):

1. One (or more) mutant subunits inhibit the activity of all other wild type subunits in a hexameric ring (Fig. 7A),
2. a mutant inhibits both of its direct ring neighbors (Fig. 7B),
3. a mutant inhibits one oriented ring neighbor (Fig. 7C),
4. defective subunits do not have any influence on the remaining subunits (not shown)
5. There is a certain threshold of defective mutant subunits (e.g. two, three etc. per hexamer) that has to be exceeded before inhibition takes place ([26]; not shown).

**Table 3:** Activity Patterns in Mixed Hexamers.

	Scheme 1		Scheme 2		Scheme 3	
configuration (see Fig. 1)	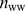 fraction of fully active wild type	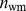 fraction of inhibited wild type	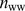 fraction of fully active wild type	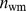 fraction of inhibited wild type	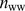 fraction of fully active wild type	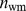 fraction of inhibited wild type
1	6/6	0	6/6	0	6/6	0
2	0	5/5	3/5	2/5	4/5	1/5
3	0	4/4	2/4	2/4	3/4	1/4
4	0	4/4	1/4	3/4	2/4	2/4
5	0	4/4	0	4/4	2/4	2/4
6	0	3/3	1/3	2/3	2/3	1/3
7	0	3/3	0	3/3	1/3	2/3
8	0	3/3	0	3/3	1/3	2/3
9	0	3/3	0	3/3	0	3/3
10	0	2/2	0	2/2	1/2	1/2
11	0	2/2	0	2/2	0	2/2
12	0	2/2	0	2/2	0	2/2
13	0	1/1	0	1/1	0	1/1
14	0	0	0	0	0	0

Numbers of wild type subunits with full and inhibited activity per hexamer.

It is relatively easy to see whether inhibition schemes (iv) and (v) apply: the ‘no inhibition scheme’ (iv) predicts that the activity per wild type subunit is unaffected by the presence of defective mutant(s); the threshold scheme (v) with 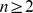 predicts sigmoidal dependencies of rates on mutant concentration that are sums of binomial probabilities, which has not been observed in any case [16], [26]. We do not elaborate on these schemes here, but focus on schemes (i) to (iii) because they produce similar inhibition patterns that are difficult to discern. 

The kinetic scheme for wild type used above required a minimum of five different reactions to account for hexamer formation. The inclusion of mutant protein in the 1–2–3–6 or 1–2–4–6 assembly pathways increases the number of possible reactions tremendously. Each reaction in figure 2 C and D can occur with any configuration of wild type-mutant oligomers. Furthermore, the two reaction partners can combine in two orientations (at the left-hand or right-hand side). For example, tetramer formation according to the 1–2–4–6 pathway from two dimers can occur as

WW+WW WWWW,

WW+WM WWWM or WMWW,

WW+MW WWMW or MWWW,

WW+MM WWMM or MMWW,

WM+WM WMWM,

WM+MW WMMW or MWWM,

WM+MM WMMM, or MMWM,

MW+MW MWMW,

MM+MW MMMW or MWMM and

MM+MM MMMM.

In principle, each of these reactions could have a unique forward and a unique reverse reaction rate. From the molecular perspective, however, it seems reasonable that the rates depend on the interface neighbors: for example, it is reasonable that the reaction WW+MM has the same rate as MW+MM because the new interaction is the same (namely XW> MY, X and Y either wild type or mutant). In our simulations, we therefore assumed identical rates for reactions leading to identical subunit interfaces. As given in the example above, we considered all permutations and all possible reactions between intermediate multimers, but left the simulation otherwise unchanged. 

**Figure 8:**
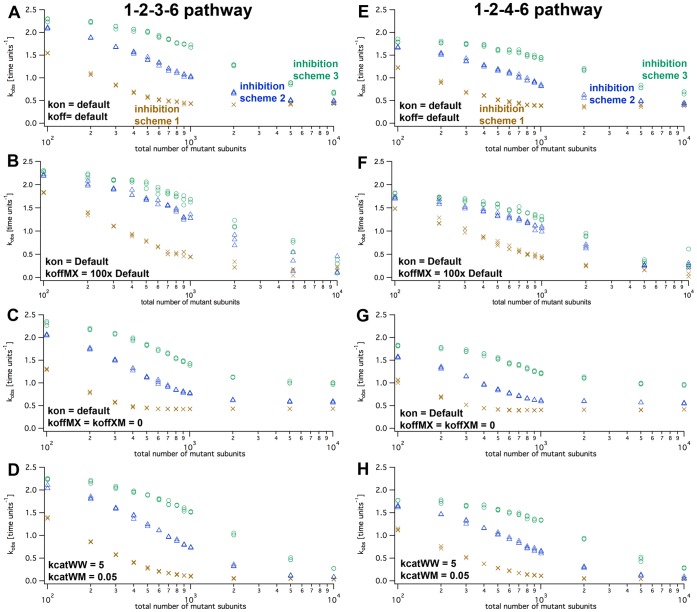
Inhibition of wild type activity by mutants. The graph plots the simulated turnover rates, kobs, as functions of mutant molecule numbers (nWt = 1000). The simulations for panels A–D used the 1–2–3–6 assembly pathway, E–H the 1–2–4–6 modelpathway. The different inhibition schemes are indicated as amber crosses (scheme 1), blue triangles (scheme 2) and green circles (scheme 3). The parameters for wild type-mutant interactions were varied as indicated and described in the text. koffXM and koffMX signify the dissociation rates of a mutant (M) with any neighbor (X) in either left or right direction.

The simulation ran stable for both assembly pathways (1–2–3–6 and 1–2–4–6) and all three inhibition schemes, and produced similar output for repeated runs with identical parameters. Increasing mutant numbers at a constant number of wild type subunits (typically, 1000) showed that the 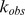 (calculated per wild type molecule) decreased in a continuously falling way (Fig. 8A and E). When wild type and mutant subunits were set to default rates for wild type and mutant, inhibition schemes 1 (Fig. 7A; one defective mutant in a ring slows down all remaining wild type subunits to a basal level) showed the strongest response on the presence of mutant. Scheme 3 (Fig. 7C; only one direct neighbor is affected) responded to the addition of mutant subunits only at higher numbers of mutant subunits. Inhibition scheme 2 (Fig. 7B; one defective mutant slows down its direct neighbors to a basal level) was intermediate. By eye, the half-maximal inhibition occurred between approximately 200 and 1000 mutant subunits for all schemes (Fig. 8A and E). The simulated inhibition pattern was very similar when 5000 instead of 1000 wild type subunits were used although it was approximately five times higher than the half-maximal activation constant for wild type only (Fig. 6). This indicates that the subunit exchange rate did not limit the kinetics in our default parameter set. 

As for the wild type case, we tested the effect of varied parameters for each inhibition scheme. Setting the association constants, 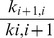, for mutant subunits to a different value than wild type by changing the forward and reverse assembly rates, as well as the variation of the catalytic constant of a wild type under the influence of a mutant, 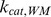, did not affect the simulation result qualitatively (Fig. 8). Upon closer inspection, qualitative differences become visible. The largest differences between the inhibition schemes occurred when 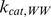 and 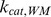 differed to a great extent (Fig. 8D and H). As intuitively expected, scheme 1 (Fig. 7A, one single defective subunit slows down all other wild type hexamer subunits to the slower 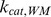 level) led to an inhibitory effect with the smallest numbers of mutant subunits (Fig. 8, yellow crosses). This effect was alleviated when the dissociation rate of mutant-wild type and mutant-mutant neighbors was assumed to be fast, meaning that the dissociation rates of neighbors with one mutant partner (termed 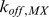 or 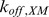, X either wild type or mutant, in figure 8) were 100-fold faster than those with wild type neighbors (Fig. 8B and F). When this rate was set to zero (i.e.: once a mutant subunit is incorporated the oligomer can only fall apart through the pathway of full assembly and ADP production), the 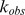 according to inhibition scheme 1 followed a steeper decrease (Fig. 8C and G). This is because in equilibrium, the majority of the wild type subunits is incorporated in oligomers together with mutants, and thus performs slow substrate turnover. In this case inhibition scheme 3 extrapolates to a relatively high 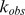 because fewer mutant subunits are available as monomers in solution to co-assemble with free wild type subunits. In addition, according to inhibition scheme 3 the effect of mutated subunits in oligomers is relatively mild. All of these features did not differ to a great extent for the 1–2–3–6 and the 1–2–4–6 assembly pathway.

### Analysis of Inhibition Experiments

One of the key questions of this study was whether it is possible to devise a simple formula that describes the inhibitory response of wild type to the presence of inactive mutants with sufficient accuracy. For other AAA ATPases, a model based on the binomial distribution of mutants in hexamers has been used frequently [26], [27], [35]. The idea is that a threshold number of mutant subunits in a hexamer leads to a complete inactivation of the remaining wild type subunits. To this end, the probability of finding a hexamer with 0, 1, 2, …, 6 mutant subunits is calculated from a binomial probability distribution at each ratio of wild type/mutant. Then, for each of the populations, a relative activity per hexamer is assigned, namely 100% if it contains less than the threshold number of mutants, and 0% else. This results in a set of 7 curves, predicting the dependence of the relative activity on the percentage of mutant present (Figure 2 in [26]). 

This model has three drawbacks: (1) the sum of wild type and mutant protein is thought to be constant, meaning that the wild type concentration is decreasing to zero towards 100% mutant. This is problematic form a theoretical viewpoint, as well as from the experimental perspective because at high mutant concentrations, the signal-to-noise ratio is poor. (2) The model does not implement allosteric effects. The concept of allostery is based on the assumption of different wild type states (T to R, in the MWC nomenclature). (3) In this model, binomial probabilities are calculated from 
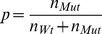
. Here, the initial, and not the equilibrium numbers are used, which are likely to differ under most conditions.

### Binomial Fitting Model

To overcome these problems, we developed novel fitting function that predicts the activity per wild type monomer as readout, allowing to discriminate between an allosterically activated or inhibited state. We also consider the possibility that wild type and mutant subunits incorporate into hexamers with different rates. Our fitting model distinguishes hexamers without mutants, and hexamers containing at least one mutant subunit. The first population comprises only wild type subunits in the fully active state (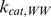; MWC nomenclature: R state), the latter contains wild type subunits in an inhibited state (with 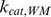; MWC: T state) and mutants (that are completely inactive). Importantly, wild type subunits only occur in hexamer configurations 1 to 13 (Fig. 1), implying that the fit function has to disregard hexamers composed of mutants only (Fig. 1, configuration 14). Then, the probability of finding a hexamer with at least one mutant subunit is (Eqn. 1):
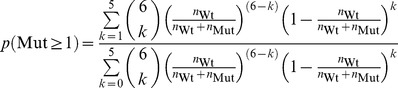




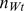
 number of wild type subunits.


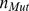
 number of mutant subunits.

The numerator reflects the number of hexamers that have 1, 2,or 5 mutant subunits, the denominator all hexamers that contain at least one wild type subunit. The probability of finding a hexamer without any mutant is (Eqn. 2):
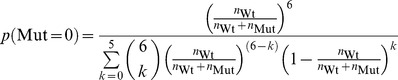



Then, the observed turnover is (Eqn. 3a):
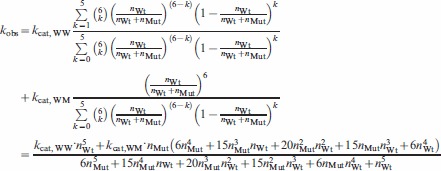




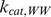
 catalytic constant of wild type under allosteric control of wild type.


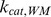
 catalytic constant of wild type under allosteric control of mutant.

To implement the possibility that wild type and mutant incorporate into hexamers with different association constants, we introduced a factor d, with which the mutant concentrations in equation 7 were multiplied (Eqn. 3b):
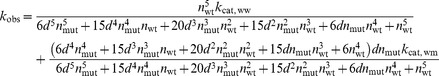



The use of 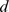 to account for different affinities between wild type and mutant subunits is a simplification that circumvents the use of non-linear equations due to the law of mass attraction. It assumes that the number of free mutant subunits in equilibrium is proportional to the number of mutants used in total. In general, this is not true. Hence, d cannot be translated easily into the ratio of affinities wild type-wild type/wild type-mutant.

### Non-competitive Fitting Model

**Figure 9:**
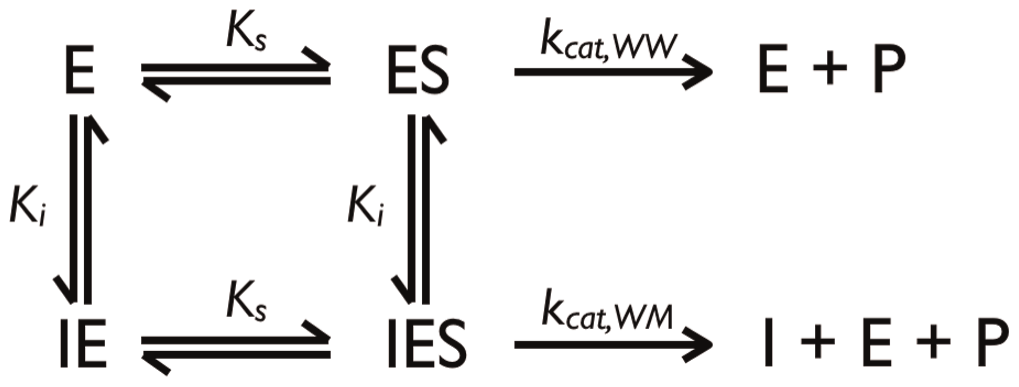
Reaction scheme for derivation of non-competitive inhibition fitting function. 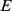
 enzyme, 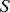 substrate, 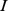 inhibitor, 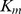 Michaelis-Menten constant, 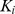 inhibition constant, 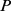 product.

As an alternative, we deduced a fit function from a non-competitive inhibition scheme (Fig. 9). Non-competitive inhibition was described by the following model. The concentrations 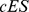 and 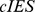 (concentrations of enzyme-substrate and enzyme-substrate-inhibitor complexes; Fig. 9) were calculated from 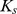, 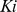, 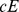, 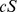 (enzyme and substrate concentrations), and cI (inhibitor concentration) by the law of mass attraction [37]. The turnover rate was defined as 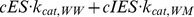. This results in the following solution (Eqn. 4):
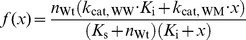



The equation is an extension of a textbook formula, with the generalization that 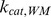 may be larger than zero [37]. It should be noted that the reaction scheme underlying this fitting function does not describe the case of co-assembly of wild type and (inhibitory) mutant subunits because in ‘classical’ models, both substrate and inhibitor concentrations are independent parameters. Therefore, in general equation 4 cannot return meaningful biochemical parameters. It turns out, however, that it fits experimental and simulated data surprisingly well. As a purely empirical comparison, we used a single-exponential fit to the data.

### Fit Functions on Simulated Data

**Figure 10:**
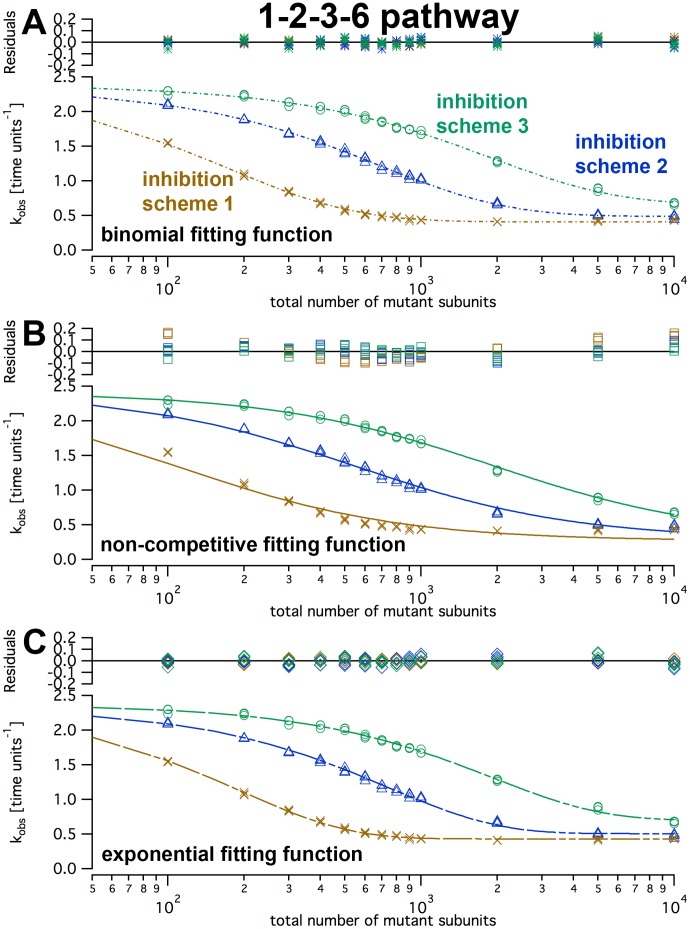
Fitting of inhibition curves. The figure shows the same simulation data points as in figure 8A (1–2–3–6 assembly pathway, default rates) with different fit functions. Curve fits to simulated data according to the 1–2–4–6 assembly pathway look essentially the same. Each panel shows the same fit function for the three inhibition schemes.

To test the feasibility of the fit functions, the simulated data from figure 8 were re-plotted with best fits for each formula (Fig. 10). Fits of simulations according to scheme 1 with the non-competitive inhibition fit function showed systematic deviations (Fig. 10B). As this formula was derived from a model that differs fundamentally from inhibition scheme 1, this observation is not unexpected. Vice versa, data sets of all inhibition schemes could be fitted by the binomial fit formula surprisingly well (Fig. 10A). This, however, does not imply that the binomial fit model describes the kinetic mechanism appropriately, as shown by the purely empirical exponential fit that matched the data equally well. 

**Figure 11:**
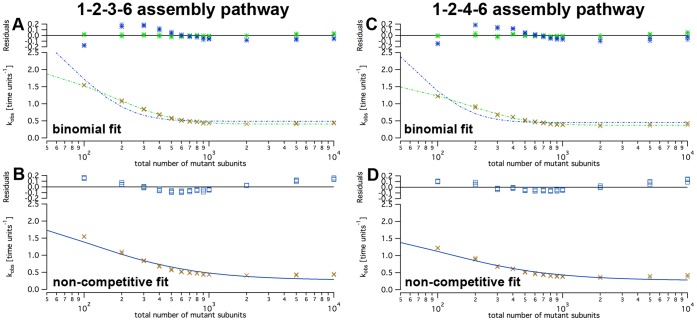
Qualities of binomial and non-competitive fit functions. The graphs show logarithmic plots of simulated data according to inhibition scheme 1 (amber crosses). Panels A and B were generated using the 1–2–3–6 assembly pathway, panels C and D the 1–2–4–6 pathway. The data were fitted to the binomial (top row, panels A and C) and the non-competitive fit function (bottom, panels B and D). Blue lines show fits with iteratively optimized kcat,WW, green lines with fixed 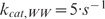.

Moreover, the binomial fit showed severe quantitative mismatches, and the fitted 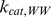 values were a factor of 2–3 too low (Tab. S1). As the maximal turnover rate of wild type is usually known from experiments in the absence of mutant, this shortcoming is particularly worrisome. We therefore repeated the fitting procedure with a fixed 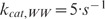. Under this constraint, the binomial fit showed a clear qualitative deviation whereas the non-competitive fit was almost identical as before (Fig. 11). With fixed 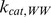 values, the fitted value of 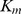 in the non-competitive scheme was very close to what was obtained previously (Fig. 4 and 5), 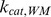 reasonably close to the value put into the simulation, and 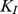 close to the point of half-maximal inhibition (Tab. S2). Considering the systematic deviations of the fit to scheme 1, the non-competitive inhibition model described the simulated data of scheme 2 and 3 with great accuracy.

### Applicability of Simulations

**Figure 12:**
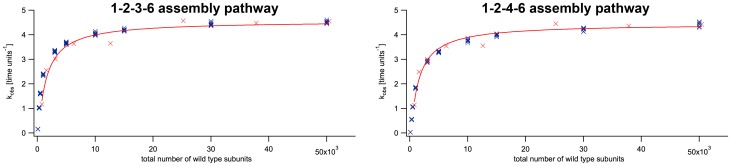
Simulated wild type data and experimental data. The figure shows the enzyme concentration-dependent ATPase activity as measured previously (red crosses [10]) in comparison to simulated data (blue crosses). The left panel shows simulated data according to the 1–2–3–6 assembly model, right 1–2–4–6.

**Figure 13:**
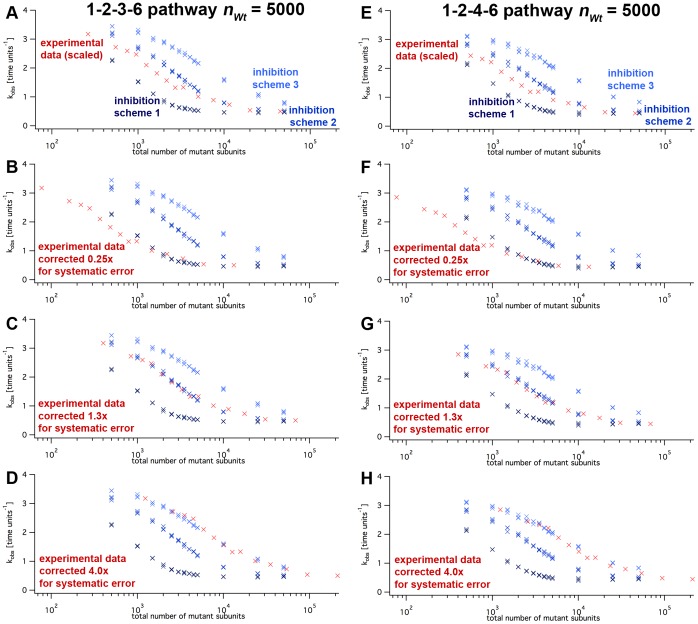
Simulated inhibition data and experimental data. The graphs show logarithmic plots of experimental data (red crosses; [10]) and simulated data (blue crosses). Panel A and E: The graphs show experimental data that was proportionally adjusted to match nWt and the uninhibited kobs (red crosses [10]), along with simulated data points (blue crosses; schemes 1–3 according to the labels). The other panels show the scaled experimental data with a correction for possible systematic errors in protein concentration determinations.

To test whether our simulation reflects the experimental data, we overlaid simulated and measured data points (Fig. 12 and 13). To this end, we scaled the experimental concentrations proportionally to the number of wild type subunits in the simulation, nWt and converted, for example, the experimental concentration of 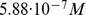 to 1000 particles. Also, as the measured 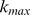 was slightly lower than simulated (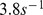 vs. 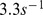 (1–2–4–6) and 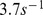 (1–2–3–6) [16]), we adjusted the 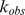 axis accordingly. 

In experiments, a strong dependence of spastin’s apparent turnover rate on the enzyme concentration was observed (Fig. 12). The activation was positively correlated with the enzyme concentration, and could be fitted by a hyperbola [16]. 

Our simulations on wild type enzyme according to the 1–2–3–6 and the 1–2–4–6 assembly pathways both showed good agreement with these data. Due to the limited number of experimental data points, which get extremely noisy at low enzyme concentrations, we cannot elucidate the assembly behavior in more detail. The empirical Michaelis-Menten curve is sufficient to describe the dependence. Our simulations also matched the experimental inhibition data with great accuracy. Figure 13 shows the calibrated experimental (red crosses), and the simulated data (blue crosses). In particular, the experimental data points fall in close proximity to simulation data obtained by inhibition scheme 2 (Fig. 13A and E). Still, none of the schemes matches perfectly. 

The situation changes if one considers that the experimentally determined protein concentrations are inherently error-prone, which may lead to systematic errors concerning the ratio of mutant and wild type protein concentrations. With 20% measurement error in each concentration, the ratio ranges between 0.67 and 1.5. When we adjusted the scaling of the experimental data manually to match either of the simulated curves best (Fig. 13B–D and F–H), we found almost perfect convergence, especially with inhibition scheme 2 (Fig. 13C and G). In this case, the systematic error correction had to be 1.3-fold, well within the estimate of the experimental accuracy. The shape of data according to inhibition scheme 1 clearly differed from experimental data (Fig. 13B and F), and the error correction had to be high (a factor of 0.25) to match the data at all. Scheme 3 resembled the scaled experimental data very closely if a 4-fold error correction was used (Fig. 13D and H). Together, these results argue that an inhibition scheme assuming interactions in two directions (inhibition scheme2) is most likely, whereas scheme 3 with interactions in one direction only is disfavored due to quantitative mismatches. The MWC-like scheme 1 is implausible because of its clear qualitative deviations.

## Discussion

AAA ATPases are a family of proteins whose members catalyze various chemo-mechanical reactions [38], [39]. The majority is active in the form of hexameric rings and displays a complex form of cooperativity [25], [40]–[43]. The large number of allelic forms of human AAA ATPases with dominant-negative pathogenic effects is known (http://omim.org/), highlighting the impact of subunit interactions and co-operativity in AAA oligomers. For the first time, we address here this problem systematically, and predict the kinetic consequences of alternative cooperative models by computer simulations. In particular, we consider assembly reactions as important steps for the steady state rate, which to our knowledge has not been done so far. 

Traditionally, co-operativity is detected in plots of catalytic rate vs. substrate concentration, where sigmoidal curves are evidence for positive co-operativity. All common explanatory models assume stable oligomeric enzymes with multiple binding sites, such as hemoglobin or the lacI protein [19], [21], [22], [44], [45]. 

Another, less conventional way to detect co-operativity is the use of hetero-oligomeric enzymes composed of intact and defective subunits. The reasoning is: If wild type subunits did not influence their catalytic behavior mutually, the addition of inactive mutant subunits would most likely have no impact on the resulting activity. Vice versa, in plots of catalytic rates (per wild type subunit) vs. inactive mutant concentration, co-operativity would be reflected by rates that depend on the mutant concentration. In fact, several publications report a decrease of the steady state catalytic turnover rate in the presence of increasing mutant concentration [16], [26]. Curiously, there is also a case published where the activity is best in 50∶50 mixtures of wild type and mutant [35]. 

Alternative concepts explaining traditional models of co-operativity have been developed in decades of work [19]–[22], [44], [46]. Models of co-operative effects induced by mutant titrations have not been worked out yet, and thus, existing publications use coarse simplifications, or even intuition. Indeed, the accurate description of these experimental setups is complicated for at least two reasons. 

One problem is that the equilibrium concentrations of enzyme monomers and oligomers cannot be calculated easily due to the fact that homo-oligomerization is a non-linear phenomenon. A frequently used approximation is that in equilibrium the concentrations of free wild type and mutant subunits are the same as the initial, total concentrations of these species [16], [27], [35], [45]. The probability of incorporating k mutant subunits into an oligomer is computed by a binomial probability function, taking the ratio of mutant subunits in total protein as the parameter p (probability of encountering a mutant). That the initial wild type and mutant concentrations are different from the equilibrium concentrations has been disregarded frequently, possibly because the calculation is complicated and requires the solution of a set of coupled non-linear equations [26], [27], [35], [45]. To overcome this problem, we devised here explicit assembly pathways, and predict specific meta-stable intermediates. This can help in the design of future single-molecule experiments that elucidate the oligomerization kinetics, a problem that has only recently been addressed experimentally [47]. 

The other problem is that modeling of co-operative effects by use of wild type-mutant mixtures requires an explicit assignment of the inhibitory mechanism. By inhibitory mechanism we mean a concise description of the inhibitory effect of mutant subunits on the other subunits of the oligomer in dependence of the configuration (Tab. 3). We think it is extremely important to consider this point because several structural investigations have shown that even homo-hexameric AAA ATPase rings may contain structurally distinct subunits [41], [48], [49]. To our knowledge, our study is the first one that systematically compares the consequences of different patterns of inhibition. 

In fact, our simulations using alternative inhibition schemes produced highly diverging curves. Scheme 1, in which one defective subunit affects the entire ring, had the strongest effect under all parameter settings. As intuitively expected, scheme 3 led to the weakest inhibition effect, and thus the highest 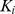. scheme 2 was intermediate. Which inhibition scheme applies to the real, experimental situation? 

We can answer this question here for spastin, for which we generated experimental data [16]. In figure 10, we compared the experimental inhibition data with our simulations. We found a strong qualitative and quantitative resemblance to inhibition scheme 2, which assumes an effect of inactive mutant subunits on both neighboring subunits. This idea is not unrealistic, and models involving allosteric coupling in both directions have been put forth for the PAN ATPase complex [50]. In fact, the model works even if allosteric coupling does not affect direct neighbors, but subunits further away. Although inhibition table 3 would differ sightly, the result would not change recognizably. 

With our simulation study we wanted to find out whether simplified kinetic models are able to explain the complicated allosteric dependencies. In the literature, a strong bias for models implementing binomial distributions prevails [26], [27], [35], [45]. However, these examples show systematic deviations between fit and data. To test whether these deviations were due to an insufficient mathematical description or whether they had a systematic cause, we developed an improved binomial fit formula accounting for the fact that (i) mixed wild type/mutant hexamers contain three levels of activity (mutant, fully active and inhibited wild type), and (ii) that wild type and mutant subunits may have different oligomerization constants (factor d in equation 3). Our model fitted experimental and simulation data qualitatively very well but failed quantitatively. The fitted 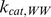 values were more than two-fold lower than the set values. When 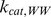 was fixed at the known number (5 s-1), the curve fit showed clear systematic deviations, even for inhibition scheme 1 (Fig. 9A and C). 

As an alternative, we used a non-competitive inhibition model, extended from textbook knowledge [37]. This model treats inhibitor binding as pseudo-first order event, which is legitimate for ligands but not for homo-oligomers [33]. Still, for many parameter sets equation 4 fitted the curves qualitatively and quantitatively with reasonable accuracy. The fitted 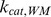 was mostly within a range of 2 from the true value but sometimes off to a much larger degree. Also, the fitted 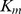 values lay close to those obtained in simulations without mutant. Interestingly, a pseudo-first order approximation came close to the fitted values but was very sensitive towards changes of any bi-molecular rate (Tab. 2). 

Together, our simulations show that ‘avalanche’ hexamer assembly pathways can describe the observed dependence of spastin activity on the enzyme concentration. Moreover, these simulations predict a negative co-operativity of inactive mutant subunits. Naively, we expected to see the best agreement between simulation data according to inhibition scheme 1 and the binomial fitting function, and inhibition scheme 3 and the non-competitive fitting function. Surprisingly, the distinction was not clear in our analyses. For experimental data, the non-competitive inhibition scheme was the only one that fitted the data with sufficient accuracy. Still, these observations show how difficult it is to conclude from global inhibition patterns of randomly mixed enzyme populations on molecular models. To circumvent these difficulties, experiments have been performed that test specific configurations (Fig. 1) by use of artificially fused AAA proteins [25]. The authors interpreted their results in the light of three models, which they called ‘concerted’, ‘sequential’ and ‘probabilistic’. The concerted model is essentially the MWC model that does not allow the simultaneous presence of R and T forms in one oligomer, with the implicit assumption that mutant subunits do not alter the rule of symmetry conservation. The sequential model describes the inhibition behavior inspired by Koshland, Nemerthy and Filmer (KNF model) who assumed an induced fit that leads to the sequential activation of subunits in the oligomer [22]. The authors excluded both of these models: They argue that their “results rule out a requirement for concerted hydrolysis in six subunits”, and their “findings were inconsistent with models requiring concerted hydrolysis by a subset of subunits” because they found hexamers that contained at least one mutant subunit but still showed full enzymatic and unfolding activity [25]. For example, a construct that separated all wild type subunits by mutants (configuration 9 in figure 1) showed wild type activity. At first sight, this seems to contradict a sequential transmission of an induced fit, as hypothesized by the KNF model. 

However, this view disregards possible differences between wild type-wild type and wild type-mutant interactions, and also possible differences between different mutants. In fact, all observations by Martin et al. can be explained by a modification of our inhibition scheme 3: If only the E185Q Walker B mutant has an inhibitory effect on the oriented, second-next neighbor, and the R370K mutant has no (or negligibly small) influence on wild type subunits, the observed rates reveal the levels of 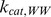 and 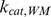 (Fig. S1). Although there may be slight adaptations necessary to fully explain all details of the study, our general claim is that it is possible to devise an inhibition scheme that fits all data, if it (i) distinguishes different allosteric activity levels in wild type subunits, (ii) clearly tabulates a network of subunit interactions, and (iii) considers that different mutants may disturb the allosteric network in different ways. These principles are in line with other studies from Sauer and co-workers that show elegantly that there is co-operativity and allosteric coupling in ClpX, and that the mutants E185Q and R370K have greatly differing phenotypes although both are inactive in homo-hexameric mutant rings [23], [24]. 

We think that the allosteric mechanism of hexameric AAA ATPases is more complicated than our current model implements. For example, there may be more than two allosteric states (wild type under influence of wild type or mutant, in our terminology, or R and T in the MWC model), and the patterns of mutual interactions may be more complicated than we implemented in our simulations. Models that view hexamer subunits in groups of three functionally coupled entities, as proposed for ClpX and PAN, are attractive and have substantial experimental support [24], [50]. In the future, we will extend our model in the light of these ideas, and link it to the force generation mechanism.

## Model and Methods

### Kinetic Monte Carlo Simulation

The simulation used here calculated the numbers of the biochemical reactants (monomeric and oligomeric enzyme species, and the ADP produced) over time based on the model and the initial values described below. The reaction was assumed to occur in an arbitrary unit volume, in which the particles were homogeneously distributed, and their encounters not diffusion-limited. The simulation implemented a Gillespie (also known as Kinetic Monte Carlo) algorithm, as illustrated in scheme 2 [34]. 

The number of simulation steps (usually 100,000 time steps), and the initial number of particles (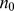, typically 1,000 to 10,000) could be adjusted. After each simulation run, the time traces were checked manually to ensure that stable equilibria had been reached. The values before equilibrium were discarded for the calculation of rates. The rate was derived from a linear fit of the ADP production over time. We implemented the simulation in Igor Pro (Wavemetrics, Portland, OR, USA), which we also used to analyze our experimental data [16]. 

The list of intermediate species is shown in figures 1 and 2, where all possible final configurations are listed (Fig. 1), and the partially assembled oligomers are embedded in the kinetic scheme (Fig. 2). The program considered the possibility that newly added subunits (or partially assembled intermediates) could join the reaction partner at either side (‘left’ or ‘right’). 

The total ATP turnover rate of wild type spastin was derived from the number of subunits present in hexamers, and their catalytic turnover rate (Eqn. 5):
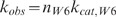




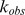
 observed turnover.


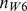
 number of wild type subunits assembled in hexamers.


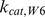
 catalytic constant of wild type spastin.

The key step in our simulation is the calculation of 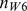. To account for the fact that hexamerization most likely does not proceed via addition of subunits one by one (1-2-3-4-5-6 pathway; Fig. 2) but via assembly of oligomeric intermediates [33], we used two models illustrated in figure 2. The assembly according to the 1-2-3-6 pathway occurs according to the following set of equations (Eqn. 6a):
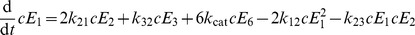


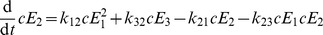


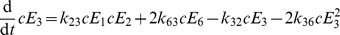


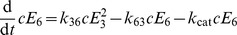



The 1-2-4-6 assembly pathway (Fig. 2) is described in the following set of differential equations (Eqn. 6b):
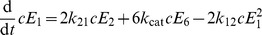


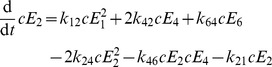


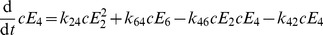


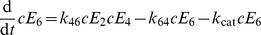



### Mutant Inhibition Simulation

To calculate the catalytic turnover of mixed wild type/mutant hexamers, each subunit in a hexamer was assigned to a specific, model-dependent 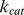, which could be either high (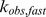) or low (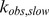). Different alternative allosteric models were formulated, and used as look-up tables for the simulation program (Fig. 1 and Tab. 3). The rationales for the competing models are given in the Results part. 

To calculate the average catalytic turnover, the simulation kept track of the number of particles in a given oligomeric state and configuration. There are four different arrangements of mixed wild type-mutant dimers (WW, WM, MW, and MM), 16 different arrangements of tetramers, and 14 of hexamers. The number of hexamer configurations is lower than that of tetramers because rotational permutations occur that lead to identical arrangements. 

The catalytic turnover numbers are always given per wild type subunit present in the simulation, not per hexamer. The apparent turnover rate per wild type subunit (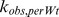) was calculated according to the following equation with values from table 3 (Eqn. 7):
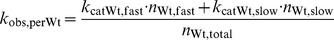
where 


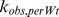
 observed apparent turnover rate, originating from wild type subunits in mixed wild type-mutant hexamers 


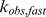
 catalytic constant of uninhibited wild type spastin (in practice identical to 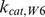) 


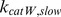
 catalytic constant of wild type spastin, inhibited by the presence of mutant subunits 


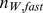
 number of wild type spastin subunits in hexamers with fast turnover 


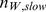
 number of wild type spastin subunits in hexamers, inhibited by the presence of mutant subunits 


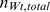
 total number of wild type subunits 

In our model, the assembly of hexamers played a central role. The program therefore allowed the variation of all kinetic rates of the 1–2–3–6 and the 1–2–4–6 pathways (Fig. 2). Both models included four forward rates and four backward rates of the dimerization reaction (W+W 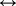 WW, W+M 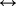 WM, W+M 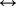 MW, M+M 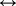 MM). The 1–2–3–6 model included 16 forward and 16 backward rates for trimer formation (XX+W 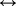 XXW or WXX, XX+M 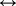 XXM or MXX; XX any of the dimer configurations) resulting in eight trimer configurations, and 64 forward and 64 backward reactions for hexamer formation, resulting in 14 different hexamer configurations (fewer than in the preceding step because of rotational symmetry) (Fig. 1). The 1–2–4–6 assembly pathway required 16 forward and 16 backward reactions for tetramer formation, resulting in 16 different tetramer configurations. The hexamerization reaction had 4 (number of dimer configurations) 16 (number of tetramer configurations) 2 (attachment of the dimer to the left or the right of the tetramer)  = 128 forwards, and 128 backward rates. 

The forward and reverse rates of all combinations between wild type and mutant intermediates were allowed to differ in the program. In practice, however, we set all rates of reactants with identical interfaces to the same value. For example, the tetramerization rate 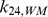 was identical for all reactions XW+MX (X any type). Otherwise, the simulations were evaluated as for wild type only.

### Analysis Methods

To analyze the simulated data sets we used the following fit functions.

#### Michaelis-Menten equation

To fit a Michaelis-Menten curve, the following equation was used (Eqn. 8 [37]):
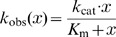


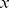
 substrate concentration

#### Pseudo-first order reaction scheme

**Figure 14:**
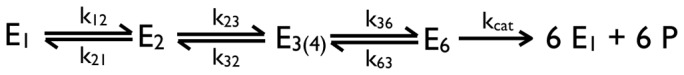
Reaction scheme for derivation of pseudo-first order approximation. The reaction scheme specifies the reactions that were used to derive the pseudo-first order formula.



The pseudo-first order reaction was derived from the following reaction scheme (Fig. 14)) and set of equations. In this scheme, only the first step is assumed to depend on the substrate concentration. Subsequent steps were treated as internal or conformational changes. This system is described by (Eqn. 9):
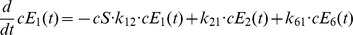


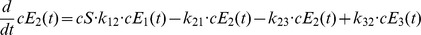


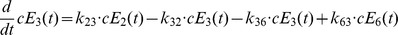


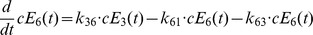


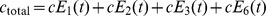


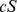
 is the substrate concentration, which in this case has to be interpreted as the concentration of the required intermediate, 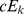. The steady state solutions are (Eqn. 10):

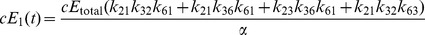


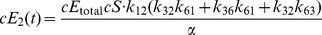


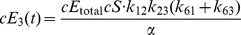


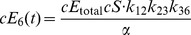


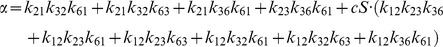



The steady state turnover is 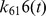, and (Eqn. 11):
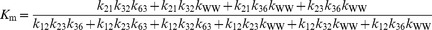



## Supporting Information

Figure S1 **Proposed allosteric network for explanation of behavior of fused ClpX wild type-mutant chimera.** Data taken from [25].
(TIF)

Table S1 **List of fitted parameters using different fit functions (all parameters free in iteration).**
(XLS)

Table S2 **List of fitted parameters using different fit functions (kcat,WW fixed to preset value).**
(XLS)
