# Supplementary figures and images for: Computer Simulation of Assembly and Co-operativity of Hexameric AAA ATPases
Source: PLoS One. 2013 Jul 15;8(7):e67815. doi: 10.1371/journal.pone.0067815 (PMC3711915; doi:10.1371/journal.pone.0067815)

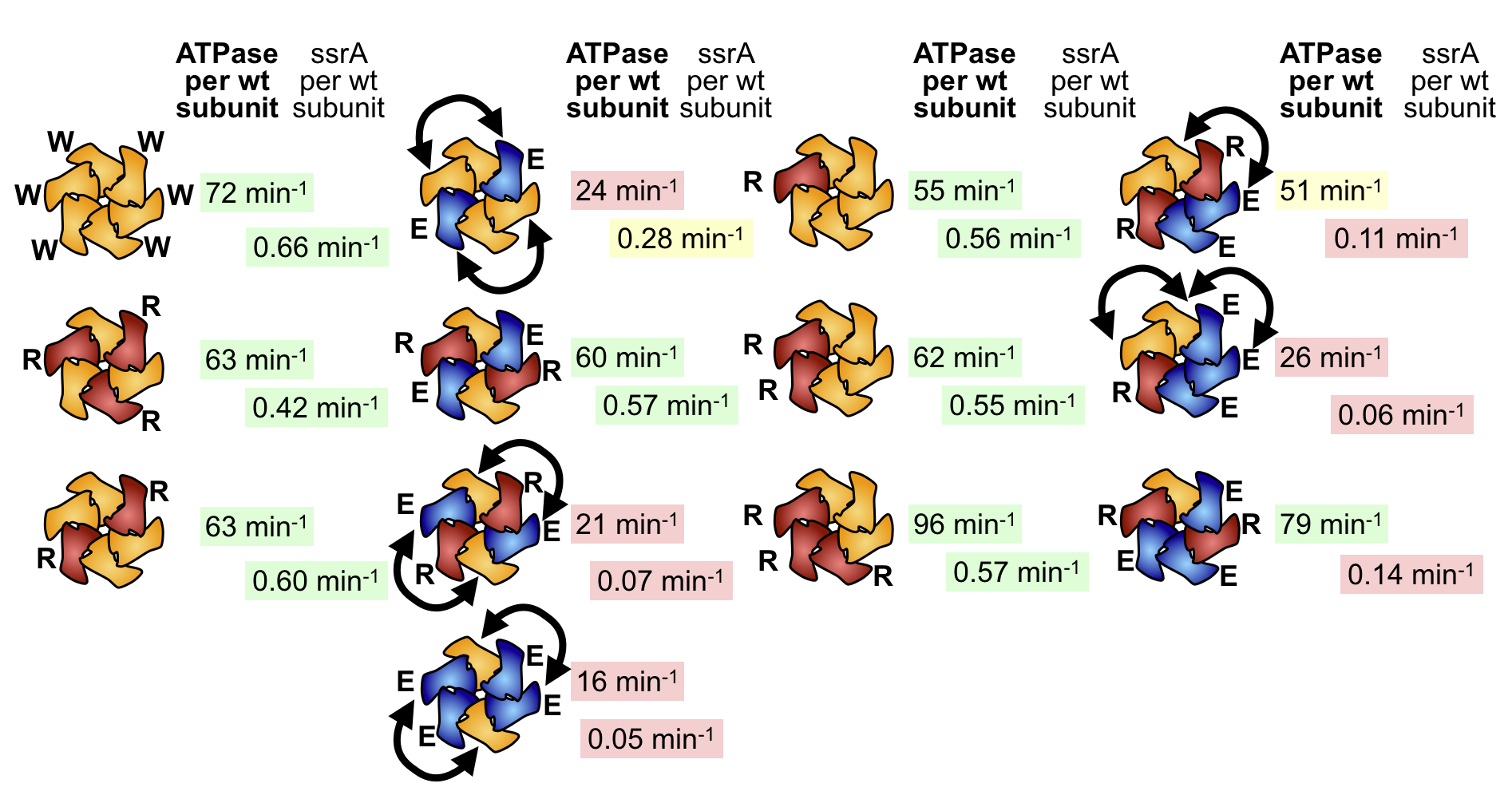

Supplement: Figure S1 — Proposed allosteric network for explanation of behavior of fused ClpX wild type-mutant chimera. Data taken from [25]. (TIF) [file pone.0067815.s001.tif]
